# Structural evidence for the role of polar core residue Arg175 in arrestin activation

**DOI:** 10.1038/srep15808

**Published:** 2015-10-29

**Authors:** Joachim Granzin, Andreas Stadler, Anneliese Cousin, Ramona Schlesinger, Renu Batra-Safferling

**Affiliations:** 1 Institute of Complex Systems (ICS-6), Structural Biochemistry, Forschungszentrum Jülich, 52425, Jülich, Germany; 2 Jülich Centre for Neutron Science (JCNS-1) & Institute for Complex Systems (ICS-1), Forschungszentrum Jülich, 52425, Jülich, Germany; 3 Institut für Experimentalphysik, Freie Universität Berlin, 14195 Berlin, Germany

## Abstract

Binding mechanism of arrestin requires photoactivation and phosphorylation of the receptor protein rhodopsin, where the receptor bound phosphate groups cause displacement of the long C-tail ‘activating’ arrestin. Mutation of arginine 175 to glutamic acid (R175E), a central residue in the polar core and previously predicted as the ‘phosphosensor’ leads to a pre-active arrestin that is able to terminate phototransduction by binding to non-phosphorylated, light-activated rhodopsin. Here, we report the first crystal structure of a R175E mutant arrestin at 2.7 Å resolution that reveals significant differences compared to the basal state reported in full-length arrestin structures. These differences comprise disruption of hydrogen bond network in the polar core, and three-element interaction including disordering of several residues in the receptor-binding finger loop and the C-terminus (residues 361–404). Additionally, R175E structure shows a 7.5° rotation of the amino and carboxy-terminal domains relative to each other. Consistent to the biochemical data, our structure suggests an important role of R29 in the initial activation step of C-tail release. Comparison of the crystal structures of basal arrestin and R175E mutant provide insights into the mechanism of arrestin activation, where binding of the receptor likely induces structural changes mimicked as in R175E.

Members of arrestin family play a central role in the regulation of signaling and trafficking of G protein-coupled receptors (GPCRs)^1^,^2^. The multistep process involves a rapid phosphorylation of the receptor protein followed by high affinity binding of arrestin that sterically blocks the interaction between receptor and G-protein, leading to the signal termination. A widely studied system to investigate the molecular mechanism of GPCR-arrestin interaction is the visual signaling cascade. Visual arrestin-1 (arr-1) is proposed to exist in basal or inactive form (Fig. 1a) that gets activated upon interaction with light-activated, phosphorylated rhodopsin (P-Rh*). Arr-1 preferentially and selectively binds P-Rh*, although it also binds weakly to dark P-Rh, as well as light-activated rhodopsin (Rh*). Previous mutagenesis reports have identified polar core residues in arrestin (R175, D30, D296, D303, R382) which when substituted result in mutants that can bind to light-activated rhodopsin independent of phosphorylation^3^,^4^,^5^. The respective residues are involved in the formation of an extended hydrogen bond network embedded between the two domains of arrestin that is structurally conserved in arrestin family (Fig. 2a). Here, residues R175 and D296 form the central salt bridge in the polar core and charge reversal of these residues results in arrestins that bind to the non-phosphorylated, activated receptor^3^,^5^,^6^. A combination of the reversed charges (R175E + D296R) restores the selectivity towards P-Rh*, that led these authors to propose R175 as the phosphate sensor residue where disruption of the polar core via receptor-attached phosphates is required to convert arrestin into the so-called ‘high-affinity’ state. Another interaction that requires breaking up for arrestin activation is the so-called three-element interaction between β-strand I, α-helix I (in the N-domain), and the C-tail (Fig. 1a)^7^. In basal state, the C-tail folds back onto the N-domain in an extended conformation, closely interacting with the two elements and ending on the top of the polar core^8^,^9^,^10^,^11^,^12^. Mutagenesis of residues mediating the three-element interaction also yield arrestins that show dramatically lowered selectivity towards P-Rh* and are active^7^. Thus, an intact polar core and the three-element interactions are the constraints that keep arrestin in its inactive state.

High-resolution structures are available of several members of the arrestin family in the basal (inactive) state^8^,^9^,^10^,^11^,^12^. Recently, crystal structures of the ‘pre-active’ splice variant p44^13^,^14^ and of β-arrestin bound to a GPCR phosphopeptide have been published^15^. In the presented work, we made a point mutation R175E in the full-length bovine arr-1 (aa 1–404, *PDB ID* 3UGX) background using site-directed mutagenesis. The purified mutant protein R175E was subjected to crystallization studies and small-angle X-ray scattering (SAXS) measurements in solution. Compared to the inactive arr-1, crystal structure as well as SAXS solution studies of the pre-active mutant R175E reveal remarkable conformational differences described below.

## Results and Discussion

### Crystal structure

**Figure 1:**
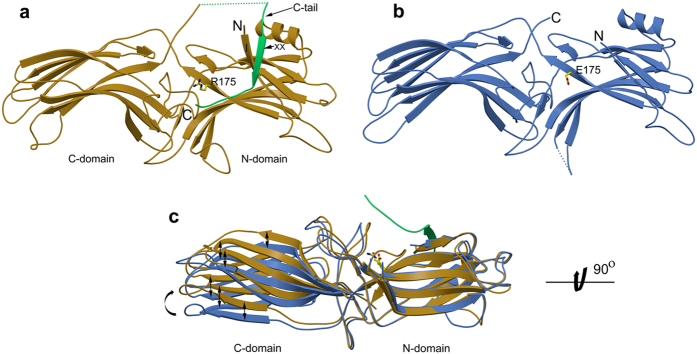
Comparison of overall conformations between arr-1 and R175E. (**a**) Ribbon representation of arr-1 (*PDB ID* 3UGX, molecule A) where C-tail is highlighted in green and the missing residues 360–370 are shown as dotted line (**b**) R175E (**c**) superposition of N-domains of arr-1 (gold) and R175E (blue) shows a 7.5° rotation in C domain of the mutant arrestin. Double-headed arrows indicate the rotation shift in the individual β-strands. Compared to panels a and b, the view here is rotated by 90° along the horizontal axis with the reader’s view upon receptor-binding concave surface. Arginine and mutated residue glutamic acid at position 175 are shown in stick model.

The mutant arrestin R175E was expressed in *Saccharomyces cerevisae* F11 α strain and purified as described in methods. R175E crystallized in space group P2_1_2_1_2_1_, with one molecule per asymmetric unit. Detailed statistics on data collection and refinement are documented in Table 1. The structure was solved by molecular replacement method as described in methods, which could be refined up to 2.7 Å resolution. The final monomer model comprises residues 11–360 (Fig. 1b). Residues 1–10 (N-terminus), 69–75 (loop V-VI or finger loop) and 361–404 (C-terminal residues including the C-tail) could not be traced in the electron density maps and thus are likely to be disordered. The mutation of residue 175 from arginine to glutamic acid (R175E) is verified in the electron density map of the mutant crystal structure (Supplementary Fig. S1). Binding characteristics of the purified R175E protein was examined for its property to bind different forms of rhodopsin using the pull-down binding assays in comparison to the wild type arr-1. In agreement with the previous reports^4^,^16^, the mutant arrestin R175E is pre-activated as it shows binding to P-Rh*, P-Rh and Rh* (Supplementary Fig. S2).

The overall structure of R175E core is similar to the previously reported arrestin structures comprising the typical bilobed structure with N- and C-domains, each consisting of eight stranded β-sandwich. A single α-helix is present in the N-domain and the polar core residues are located in the fulcrum of the domain interface (Fig. 1b). Predicted as the ‘phosphosensor’ and central residue of the hydrogen bond network in the polar core region, mutation of R175 to E is expected to have a severe impact on the structure.

### Polar core

**Figure 2 f2:**
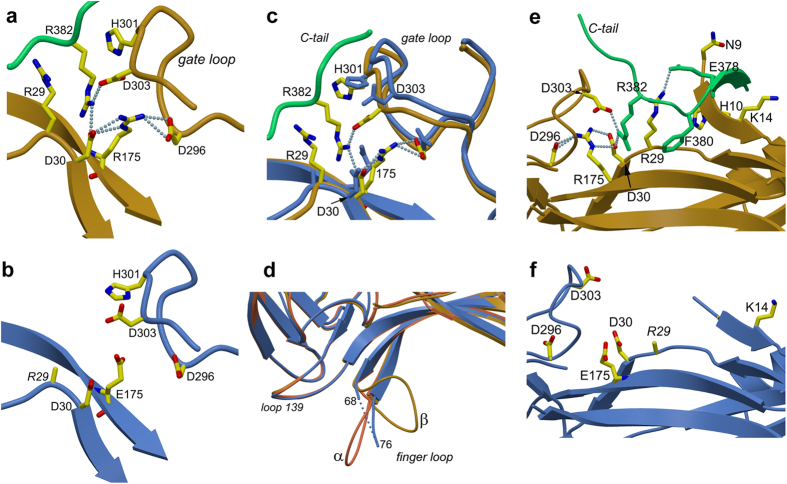
Structural differences between arr-1 (*PDB ID* 3UGX, molecule A) and R175E. (**a**) The hydrogen bond network in arr-1 around Arg 175 linking residues (shown in stick model) from polar core, gate loop and the C-tail (shown in green). Hydrogen bonds are shown as blue dotted lines (for clarity, h-bonds shown are ≤3.2 Å) (**b**) R175E polar core region shows a complete collapse of the hydrogen bond network (**c**) superposition of arr-1 and R175E showing differences in the polar core region. Gate loop in R175E shows an outward shift and the residues of this loop show different rotamers. Additionally, E175 is exposed, and the side chain of R29 is truncated beyond Cβ position due to missing electron density. (**d**) Superposition of arr-1 and R175E showing the finger loop and loop 139. Both, α (extended, as in molecule B in *PDB ID* 3UGX, orange) and β (bent, as in molecule A in *PDB ID* 3UGX, gold) conformations are seen in arr-1. In R175E, no electron density could be traced for residues 69 to 75 of the loop that is likely disordered (shown in dotted blue line). (**e**) Anchoring of C-tail and three-element interaction in arr-1 showing hydrogen-bond interactions between residues from the polar core region, the N-terminus and the C-tail. (**f**) In R175E, showing absence of hydrogen bond interactions between the polar core residues and the C-tail as well as surface exposure of the residues. Also, note the antiparallel positioning of the C-tail in panel e where R382 is the terminal residue in arr-1 crystal structure to participate in the polar core network. Disruption of polar core in R175E releases R382, a major constraint that once released leads to enhanced C-tail flexibility. Energetically, this in turn can initiate the disruption of further H-bond interactions (between the C-tail residues 380, 379, 378 with the central residue R29; Supplementary Table S2), causing the R29 side chain to disorder.

The region is composed of several charged residues that form a hydrogen bond network and is highly conserved among members of the arrestin family. R175 forms a central salt bridge, restrained by D296 and D30 (Fig. 2a). The hydrogen bond network in the polar core collapses in R175E, where all the hydrogen bonds with residue E175 are broken (Fig. 2b), increasing the side chain B-factor by ∼50% (see Supplementary Table S1 for the list of hydrogen bonds in different arrestin structures from pdb database). However, the backbone interactions E175-N…O-R29 and E175-O…N-Y31 involving the Cα, as well as the secondary structure elements and positioning of the polar core residues E175 (R175 in arr-1), D30, D296 and D303 are similar in both arr-1 and R175E with an rmsd of ∼1.24 Å^17^. Primary residues contributing to the polar core are localized on the β-strands (D30, R/E175) and the gate loop (D296, D303) that we examined next.

### Gate loop

In the crystal structures of arr-1 and other arrestins in basal state, the gate loop residues D296, D303 and N305 form a hydrogen-bonding network with R175 (numbering refers to arr-1). In contrast, this network does not exist in R175E (Fig. 2b[Fig f2]c). Superposition of R175E on arr-1 shows that the gate loop is pushed away from the central residue 175 of the polar core (Cα R/E 175E – Cα D303 distance is 10.6 Å and 12.2 Å in arr-1 and R175E, respectively) (Fig. 2c). Secondly, side chains of several residues in the loop show different rotamers, mostly facing away from the center. An outward shift of the loop and lack of the hydrogen bonding network opens the polar core cleft slightly. Side chain of E175 also faces away from the center and is now exposed. Not surprisingly, the Cα position of residue 175 remains unchanged, which is located relatively fixed on the β-strand X (residues 169–178) (Fig. 1).

On the receptor-interface on concave side, loops containing residues 139, 157 and 344 correspond to the ‘plastic’ regions of arr-1 that show high flexibility in both arr-1 and P-Rh* complex^18^, as well as in distinct conformations in monomers of crystal tetramer (Supplementary Fig. S3)^19^. These loops in R175E show conformations similar to that in arr-1 (Fig. 2d). Several of the loops have been previously reported to play a role in receptor binding^7^,^20^. Specifically, these include the finger loop (residues 68–79), loop 139 or middle loop (residues 133–142), and lariat loop (283–305, including the gate loop residues 296–305 described above).

### Finger loop

Significance of the finger loop in the arrestin-receptor binding interface has been established through biochemical and structural data^7^,^18^,^19^,^21^. Located between the two domains, the loop is anticipated to bind within the crevice that opens up on the cytoplasmic face of the receptor during TM6 and TM5 movements^22^. Conformational flexibility in the finger loop (residues 68–79) has been proven to be essential for arrestin to adopt high affinity binding to the receptor^18^,^23^,^24^,^25^,^26^,^27^. In arr-1 structure, this loop attains two conformations: extended as in α-conformer, and folded-in towards the concave surface of the N-domain as in β conformation^8^,^10^,^13^. In R175E, residues 69–75 in the loop could not be traced in the electron density map and are likely to be disordered (Fig. 2d). This is unexpected as residues of the finger loop are not in direct contact with the polar core. Additionally, the loop is solvent-exposed and shows no crystal contacts. Just recently, crystal structure of rhodopsin-arrestin complex has been published where the arrestin finger loop clearly adopts a α-helical conformation and forms the primary interface between the two proteins where it interacts extensively with the C-terminus of TM7, the N-terminus of helix 8 and the intracellular loop 1 (ICL1) of rhodopsin^28^ (Supplementary Fig. S4). The α-helical conformation of the finger loop peptide analogues bound to rhodopsin was shown previously by solution NMR^29^ and crystallography^22^. Both, missing electron density as seen in R175E mutant and conformational variability of the finger loop as in various crystal structures are suggestive of the flexible nature that is likely to reflect the activation dependent changes^13^,^14^,^15^, as now evident from the rhodopsin-arrestin complex structure^28^. Interestingly, a common feature seen in the crystal structures of pre-activated arrestins, the rhodopsin-arrestin complex and the R175E mutant is also disruption of the polar core (Table S1). Both, disordered finger loop and a completely disrupted polar core in R175E are thus consequences of the point mutation.

### Anchoring of the C-tail and three-element interaction

The C-terminal residues 371–386 in the basal state structure are folded onto the polar core region, blocking binding of the phosphorylated receptor. Anchoring of the C-tail mainly takes place via interaction between residues of the C-tail (most conserved are R382, F380, E378), the polar core (D30, D303, R175), the arginine switch (R29) and the N-terminal β-strand I (here C-tail residues form an anti-parallel β-strand XX) (Fig. 2e) (Supplementary Table S2). As mentioned elsewhere, the basal state of arr-1 is stabilized mainly by the polar core and the three-element interaction, where the latter includes residues from β-strand I, α-helix I, and β-strand XX (Fig. 1a).

Compared to arr-1, a major structural difference in R175E is the absence of electron density for C-terminal residues (361–404) that includes the C-tail residues. The region, in general, is flexible^30^ as residues 362–370 and its equivalents could not be resolved in all previously published arrestin structures^8^,^9^,^10^,^11^,^12^. The disordered residues form the linker region connecting the C-domain and the C-tail, where the latter binds along the N-domain and is the only ordered part of the C-terminus in the crystal structures of full-length arrestins (Fig. 1a, 362–370 are shown in dashed line). As the point mutation was introduced in the wild type background, we first checked for the presence of C-terminal residues in the crystals (Supplementary Fig. S5). SDS-gel and mass spectrometry (MALDI-TOF) analysis (data not shown) of R175E crystals show the presence of full-length protein, with no indications of proteolysis. Additionally, results of size-exclusion chromatography (Supplementary Table S3) and SAXS analysis of arr-1, p44 and R175E are also suggestive of the presence of R175 in full-length in solution.

Release of the C-tail plays an important role in initiation of arrestin activation that precedes high affinity binding of the phosphorylated C-terminus of receptor^7^,^21^,^30^,^31^,^32^. In the crystal structure of arr-1, residue R382 in the C-tail shows hydrogen bonding with residues R29, D30, D303 and L173 in and around the polar core (Fig. 2e). Of these residues, R29 is hydrogen bonded to E378, E379, F380 and R382, interactions that are majorly responsible for anchoring of the C-tail as in the basal state. Interestingly, the side chain of R29 is disordered in R175E as it could not be traced in the electron density map beyond the Cβ position (Fig. 2f). The dynamic nature of the R29 side chain is to be seen in the reported arrestin structures, where it adopts different rotamers (Supplementary Fig. S6). In our previously reported structure of the truncated splice variant p44 arrestin, R29 points ‘in’ the polar core where it interacts with D30 and R175, taking place of the missing C-tail residue R382^13^. In contrast to the side chain flexibility of R29, the backbone interaction R29-O…N-E175 is conserved in all arrestin structures including R175E structure, suggesting importance of the close proximity of the two residues in arrestin function. Recently, R29 was suggested to control the C-tail exchange mechanism where the C-tail of arrestin is released with several charged residues exposed for binding of the phosphorylated C-terminus of receptor^33^. These authors performed an extensive mutagenesis study, where mutant R29A was shown to be the weakest binder to P-Rh*. Furthermore, the equivalent residue of R29 in β-arrestin is in direct contact with the GPCR phosphopeptide in the recently published crystal structure of β-arrestin-1-peptide complex, confirming this region as binding site for the phosphorylated receptor^15^. No structure coordinates were reported for several C-terminal amino acids (362–393, β-arrestin-1 numbering) in the same structure and are presumably flexible. Likewise, no electron density could be detected for the C-terminal residues (362–392) in the crystal structure of rhodopsin-arrestin complex^28^. In R175E, collapse of the hydrogen bond network in the polar core (R382 is no longer anchored here) is likely to cause release of the C-tail, breaking the interactions with R29, consequently increasing the side chain flexibility (Fig. 2f).

### Small angle X-ray scattering (SAXS)

**Figure 3:**
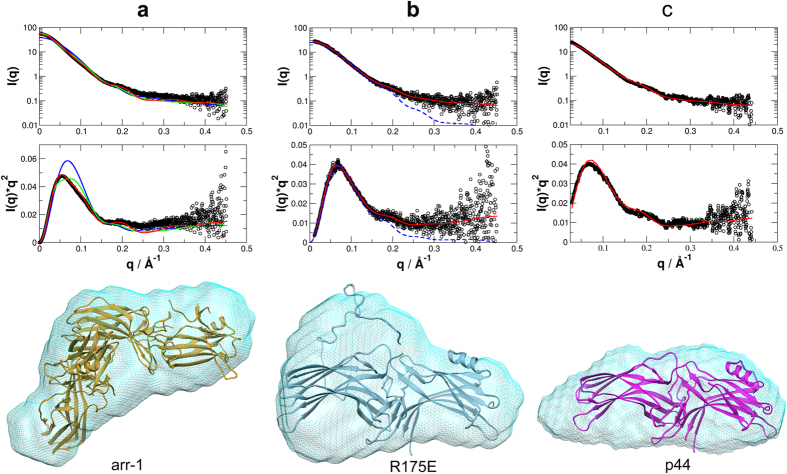
Characterization in solution by small angle X-ray scattering (SAXS). (**a**) arr-1, (**b**) R175E and (**c**) p44 (as control). *Upper panels* show experimental SAXS curves (black circles) overlaid with the theoretical scattering curves calculated from the respective crystal structures. In (**a**), X-ray crystal structure (*PDB ID*: 3UGX) used are of arr-1 monomer (blue line: molecule A), and two possible dimers (green line: molecule A and B; red line: molecule A and D); in (**b**) crystal structure of the R175E monomer including the flexible C-terminus (red line), and without the flexible C-terminus (dashed blue line); and in (**c**) crystal structure of p44 (*PDB ID*: 3UGU) monomer (red line). *Middle panels* show the respective experimental data and the fits in Kratky-plots. *Lower panels* show the respective *ab initio* models (cyan, shown in mesh) determined by SAXS. Ribbon structures of the respective crystal structures: Gold, dimer with molecules A and D (*PDB ID*: 3UGX) in (**a**); blue R175E in (**b**), and magenta p44 (*PDB ID*: 3UGU). Orientation in (**b**) and (**c**) are as in Fig. 1. The dimer of arr-1 in (**a**) is positioned such that molecule A on the top is oriented as in Fig. 1c.

SAXS analysis was performed to determine the oligomeric state and low-resolution shape of R175E in solution. For comparison, arr-1 and the truncated splice variant p44 were measured under identical conditions as described in methods. The molecular mass determined from the Porod volume and *ab initio* modeling is 43.5, 45.4 kDa and 81.2 for R175E, p44, and arr-1, respectively (The calculated molecular mass from the amino acid sequence for R175E, p44 and arr-1 dimer is 47.1, 41.1, and 94.2 kDa) (Fig. 3). As indicated from the analytical size-exclusion chromatography (Supplementary Table S3), SAXS results confirm that the wild type arr-1 is dimeric, with the best fit comprising A/D chains of the crystal structure PDB ID 3UGX as judged by the χ-value, the *R*_*G*_, and the *ab initio* model. (χ-value is defined as 
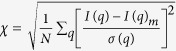
, where *I*(*q*) and *σ*(*q*) are the measured intensities and errors, respectively, *I*(*q*)_m_ are the calculated intensities from the structural model, and *N* is the number of data points). The measured Guinier radius *R*_*G*_ of arr-1 is 37.4 ± 0.1 Å. The *R*_*G*_ of 3UGX monomer, the 3UGX A/B chains and the 3UGX A/D chains is 27.9, 41.6 and 35.5 Å, respectively. The fits yielded corresponding χ-values of 32.2, 10.3, and 5.1 for the monomer, the A/B dimer, and the A/D dimer, where a lower value of χ indicates a better fit (Fig. 3a).

In contrast, mutant R175E and p44 are monomeric with experimentally determined *R*_*G*_ values of 28.8 ± 0.2 Å and 31.9 ± 0.2 Å. The corresponding *R*_*G*_ values calculated from the crystal structures are 28.0 and 27.9 Å. It is interesting that the crystal structures of both proteins contain one monomer per asymmetric unit current work,^13^. In case of R175E, the data fits best with the C-tail kept flexible (χ = 0.67 for the structure with flexible C-tail vs. χ = 1.70 for the crystal structure only) (Fig. 3b). The calculated *R*_*G*_ of 29.1 Å of the structural ensemble with flexible C-tail is also closer to the measured *R*_*G*_ of 28.8 Å. This is in agreement with the *ab initio* model of R175E, which is more extended in the region of the flexible C-tail when compared to that of p44 (Fig. 3b,c).

### Structural differences between R175E and the ‘active’ crystal structures

One of the properties reported in the recently published crystal structures of arrestin in ‘active’ state is a 21° rotation between N and C domains^14^,^15^. Compared to arr-1, R175E shows a 7.5° rotation (Fig. 1c). A possibility of rotation between the domains was previously suggested in molecular modeling^34^ and truncation experiments in the inter-domain hinge region^35^. Relative movement of domains is supportive of the ‘clam-shell’ model where these transitions are required for arr-1 to bind the receptor in ‘high-affinity’ state^7^. In contrast, using site-directed spin labeling and double electron-electron resonance, Kim *et al*. measured long-range (~17–60 Å) intramolecular distances in arr-1 in solution in the presence of P-Rh* to show that the relative position of N and C domains remains largely unchanged^19^. Similarly, no evidence of domain movement could be seen in non-visual arrestins^31^, thus working against the ‘clam-shell’ model. An important clue comes from the first crystal structure of rhodopsin-arrestin complex^28^ that reveals a ~ 20° rotation between N and C domains, as seen previously in the two ‘activated’ arrestins^14^,^15^. The domain movement opens a cleft in arrestin to accommodate the short helix formed by the second intracellular loop of rhodopsin (Supplementary Fig. S4). The physiological relevance of the structural model of the complex was extensively validated by the authors using double electron-electron resonance, hydrogen-deuterium exchange mass spectrometry, cell-based rhodopsin-arrestin interaction assays and site-specific disulfide cross-linking experiments. The domain movement seen in R175E and the three activated arrestins is suggestive of its role in arrestin activation mechanism that may apply to all members of arrestin family.

Other structural differences between R175E and the active states are seen in the gate, finger and the middle loops (loop 139)^14^,^15^ (Supplementary Figs S4, S7). Role of the 139 loop has been previously proposed in stability and binding selectivity of arr-1^36^. Using double electron-electron resonance (DEER) data, large conformational changes in the loop 139 were reported in arr-1^19^, but not in β-arrestin where the loop was suggested to be highly flexible in both the basal and active states^31^. Furthermore, functional mapping of arr-1 at single amino acid resolution showed no significant binding effects to Rh* when residues of this loop were mutated to alanine^33^. However, similarity of the middle loop conformation in the pre-activated arrestins and in the rhodopsin-arrestin complex as well as its position at the interface in the later structure confirms its role in receptor binding. As shown in supplementary Fig. S4, in order to bind rhodopsin, the loop as in the basal state needs to move up to avoid the steric clashes. This in turn will have an influence on the gate loop conformation as seen for the three ‘activated’ arrestin structures (Supplementary Fig. S7).

Release of C-tail is predicted to expose the residues of gate loop (296–305) for interaction with the activated receptor^7^. In R175E, the C-tail is disordered exposing the lariat loop residues, and yet the overall conformation remains similar to that of arr-1. The loop is mostly stabilized by hydrogen bonding within intra-loop residues (also see Table S1 for a detailed list of hydrogen bonds in the polar core for various arrestin structures deposited in pdb database). Surprisingly, the gate loop residues show no contact with rhodopsin in the complex structure^28^. As mentioned elsewhere, conformational flexibility in the finger loop residues has been established both structurally and biochemically. Residues of the finger loop are disordered in R175E. It is likely that in the pre-activated state the loop becomes highly flexible ‘searching’ for the correct binding site in the receptor, which is stabilized once bound to the receptor as seen in the rhodopsin-arrestin crystal structure where it adopts a α-helical conformation^28^. Notably, crystals of pre-activated p44 and β-arrestin with the finger loop in extended conformations were obtained in the presence of the ligands opsin and phosphopeptide V2Rpp-synthetic antibody fragment Fab30, respectively^14^,^15^. Crystallization of R175E was performed without any ligand. The 20° rotational change in domains as well as significantly different loop conformations seen for the three above-mentioned loops in case of ‘pre-activated’ arrestins^14^,^15^ is likely induced by the presence of ligand protein/peptide, as revealed in the crystal structure of rhodopsin-arrestin complex^28^. Additionally, the gate loop residues in the β-arrestin–peptide complex show interactions with the peptide bound to arrestin as an antiparallel β-strand replacing the C-tail^15^.

### Conformation of R175E arrestin: basal, pre-active or active?

In the current model of arrestin activation, interaction of receptor-attached phosphates with polar core residues results in conformational changes including breaking of the three-element interaction and release of C-tail, allowing formation of the initial receptor-arrestin complex. Subsequently, further structural changes in arrestin cause its transition to high-affinity binding state^7^,^20^. Previous mutagenesis studies have shown that like R175E, mutation of residues in the polar core, C-tail and those involved in the three-element interaction also result in pre-active arrestins that bind light-activated rhodopsin irrespective of its phosphorylation state^3^. The most likely explanation common to these mutations is the release of a stereochemical constraint responsible to keep arrestin in its basal state. For the highly conserved ion pair R175 and D296, it was proposed that neutralization or charge reversal of the residues result in mutants that mimic the effect of phosphate groups from the receptor, allowing them to bypass the need of receptor phosphorylation^3^. Formation of this state would involve structural rearrangements in the polar core, C-tail and three-element interaction. Additionally, the authors speculated a change in position of the N- and C- domains relative to each other for arrestin to achieve the high-affinity binding state. The structure of R175E presented here is the first structure report of an arrestin with mutation in the polar core that shows a completely disrupted hydrogen bond network in the polar core (also see Table S1), a 7.5° rotation between N- and C- domains, and a ‘free’ C-tail. Furthermore, the displacement of C-tail residues causes an increase in the overall positive electrostatic potential and exposure of the key phosphor-sensor residues (Supplementary Fig. S8), allowing it to bind the receptor irrespective of its phosphorylation state as has been previously reported for the truncated arrestins^13^,^14^,^34^. As the mutations in case of other pre-active mutants of arrestin are located in the polar core, the three-element or the C-tail regions^3^, it can be speculated that the conformational rearrangements and the activation mechanism in the mutants would be similar to R175E.

Taken together, the structural features of R175E and its differences to the basal and ‘active’ states tempt us to propose that the structure presented with disrupted polar core and three-element interaction with a ‘free’ C-tail represents an intermediate activation state prior to formation of the high-affinity ligand complex^20^. Consistent to the arrestin activation model, the first step involves phophosensor interaction and the release of C-tail, breaking the three-element interaction. This is then followed by the ligand-induced conformational changes for the formation of ‘high-affinity’ state bound to the receptor. This includes a ~20° rotation movement between N and C domains, which opens a cleft between the middle loop (loop 139) and the C-loop (loop 250), allowing binding of the finger loop to the rhodopsin^28^. Additional structural changes are expected to take place for optimal binding of the receptor such as seen in the loop conformations in case of rhodopsin-arrestin^28^ and β-arrestin phosphopeptide complexes^15^.

## Methods

### Expression, purification and crystallization of R175E mutant arrestin

Recombinant mutant R175E was generated using oligonucleotide-directed mutagenesis by introducing the point mutation in arr-1 as described previously^37^. The mutagenesis primer used for the PCR amplification was: 5′-CGT TTG CTG ATC **GAG** AAG GTA CAG-3′. The resulting mutated R175E gene was verified by sequencing and cloned into the vector pYEX-BX (Clontech, Heidelberg). Large-scale expression and purification of recombinant arrestins R175E, arr-1 and p44 were carried out in *Saccharomyces cerevisiae* F11 α strain as described before^37^. Purified R175E was concentrated to a protein concentration of 13 mg/mL, and crystallization setups were performed using the vapor-diffusion method. Crystals were grown in 2-μl sitting drops (1 μl protein + 1 μl reservoir solution) against 100 mM Tris (pH 7.5) and 8–18% (w/v) polyethylene glycol 4000 at 19 °C, where the first crystals appeared within 8–10 days that were allowed to grow further for another two weeks.

### Data collection

The X-ray diffraction datasets were collected at 100 K. Prior to cryo-cooling, the crystal was soaked stepwise in reservoir solution containing up to 20% (v/v) glycerol. Native data were recorded at the beamline ID23-1 of the ESRF (Grenoble, France) tuned to a wavelength of 0.979 Å on a PILATUS 6M-F detector (DECTRIS). The data collection strategy taking radiation damage into account was based on calculations using the program BEST^38^. Data processing including reflections up to 2.7 Å resolution was carried out using XDS^39^ and AIMLESS (CCP4)^40^.

### Structure determination

The structure was determined by molecular replacement using MOLREP (CCP4) with a merged native dataset. The search model was modified from the arr-1 model PDB ID 3UGX with the point mutation E175, which was reduced to alanine for control purpose. Crystals were found to contain one molecule per asymmetric unit, corresponding to a Matthews coefficient of 2.03 Å^3^/Da and a solvent content of 39.3%. Following rigid-body refinement using the PHENIX package^41^, the model was improved in an iterative manner, including several cycles of positional, isotropic temperature factor and TLS refinement with PHENIX and manual rebuilding using the program COOT^42^. For statistics on data collection and refinement refer to Table 1. According to Ramachandran plot generated with Molprobity (PHENIX), the model exhibited good geometry with none of the residues in disallowed regions. Relative rotation between the domains in R175E and wild type arr-1 (3UGX chain A) were computed using algorithm Hingefind^43^ that resulted in an effective rotation angle of 7.53° with the relative error of 0.065%.

### Small angle X-ray Scattering

Small angle scattering was measured on the BioSAXS beamline BM29 at the ESRF^44^. The temperature was kept at 10 °C throughout the experiment. Ten individual frames with 1 s exposure time were recorded, while the samples were continuously purged through a quartz capillary. The frames without radiation damage were averaged, and contribution of the buffer was subtracted from measured intensities of the protein solution. Data were scaled by the measured protein concentrations and extrapolated to infinite dilution. Three concentration series were measured (arr-1: 1.9, 3.0 and 8.5 mg/mL, R175E: 0.4, 0.9 and 1.7 mg/mL, p44: 1.0, 2.0 and 4.6 mg/mL) in buffer containing 10 mM HEPES, 150 mM NaCl, pH 7.5.

SAXS data were analysed using the programs available within the ATSAS software package^45^. Scattering curves of the crystallographic structures were calculated and fitted to the experimental SAXS data using the computer program CRYSOL. The distance distribution function *P*(*r*) and the Porod volume of the protein were determined with the programs GNOM and DATPOROD, respectively. *Ab initio* models were generated using the DAMMIF program where twenty models were generated, averaged and the filtered model was used. The envelope function of the filtered *ab initio* model was visualized using the SITUS package^46^. The molecular mass was estimated from the excluded volume of the filtered *ab initio* model and from the Porod volume using division factors of 2 and 1.7, respectively^45^. Flexibility of the C-tail of R175E arrestin was modelled using the program EOM. A large pool of 10,000 random-like conformations of the tags and the flexible C-tail attached to the crystal structure was generated. Positions of the residues 69–75 (loop V-VI) were taken from the open α-conformation of arr-1. Consecutively, a representative conformational ensemble was selected, which best describes the measured data. In the small-angle range, no significant differences are observed between the α or the β finger loop conformation in the SAXS analysis of arr-1.

## Additional Information

**Accession codes:** The atomic coordinates and structure factors have been deposited in the Protein Data Bank under accession code 4ZRG.

**How to cite this article**: Granzin, J. *et al*. Structural evidence for the role of polar core residue Arg175 in arrestin activation. *Sci. Rep*. **5**, 15808; doi: 10.1038/srep15808 (2015).

## Supplementary Material

Supplementary Information

## Figures and Tables

**Table 1:** Data collection and refinement statistics on arrestin R175E*.

Data collection	
Radiation source	ESRF ID23-1
Wavelength (Å)	0.97902
Resolution range (Å)	41.64 – 2.7 (2.83 – 2.7)**
Space group	P 2_1_ 2_1_ 2_1_
Cell dimensions	
a,b,c (Å)	66.37, 72.31, 79.4
Total reflections	83633 (9579)
Unique reflections	10993 (1430)
Multiplicity	7.6 (6.7)
Completeness (%)	99.92 (99.91)
〈I/σ(I)〉	10.8 (2.5)
Wilson B-factor (Å^2^)	38.6
R-merge	0.217 (0.924)
R-measured	0.234 (1.005)
Refinement	
Resolution range (Å)	41.64-2.7 (2.97–2.7)**
R_work_	0.1938 (0.2742)
R_free_	0.2414 (0.3214)
No. of atoms	2677
Protein	2608
Protein residues	343
Ligands	15
Solvent	54
R.m.s. deviation from ideal	
Bonds (Å)	0.01
Angles (°)	1.01
Mean B-factor (Å^2^)	44.1
Protein	44.2
Ligands	63.0
Solvent	34.6
Ramachandran statistics (%)	
Favored regions	98
Outliers (%)	0

^*^Merge of two datasets.

^**^Values in parentheses are for the highest resolution shell.
