# Supplementary material for: Structural evidence for the role of polar core residue Arg175 in arrestin activation
Source: Sci Rep. 2015 Oct 29;5:15808. doi: 10.1038/srep15808 (PMC4625158; doi:10.1038/srep15808)
Supplement: Supplementary Information [file srep15808-s1.doc]

**Supplementary Information**

**Structural evidence for the role of polar core residue Arg175 in arrestin activation**

Joachim Granzin, Andreas Stadler, Anneliese Cousin, Ramona Schlesinger, Renu Batra-Safferling^*^

**Supplementary Figures**

**
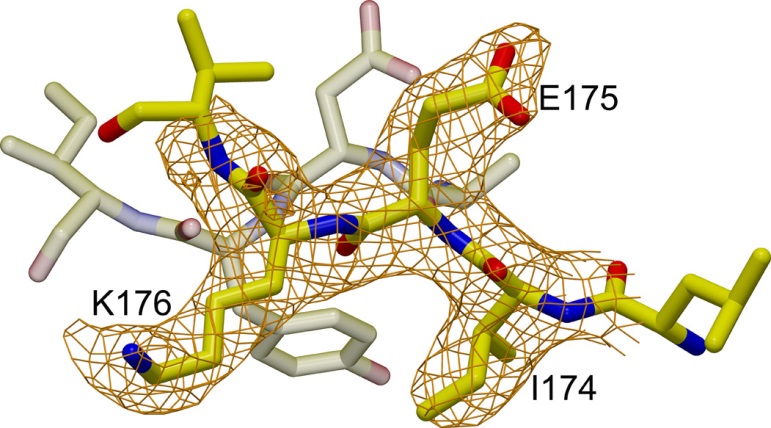
**

**Figure S1.** The electron density map of the mutated residue in crystal structure of R175E mutant arr-1. The σ-A weighted *2mFo* - *DFc* electron density map of the mutated residue E175 and neighboring residues I174 and K176 is contoured at 1.2 σ, and are shown as stick models colored by element: carbon, yellow; nitrogen, blue; oxygen, red. Residues of neighboring β-strand III are shown transparent in the background.

**
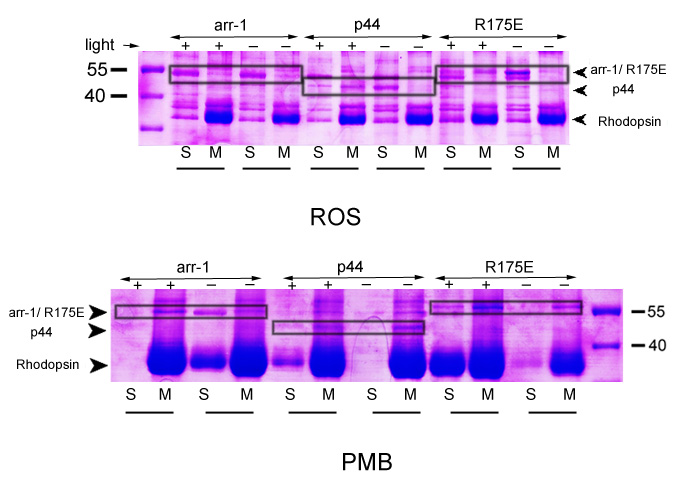
**

**Figure S2.** Effect of mutation R175E on binding properties to four functional forms of rhodopsin- P-Rh*, P-Rh, Rh* and Rh, using pull down assays. Rod outer segment (ROS) membrane, and Phosphorylated membrane (PMB) were prepared as described in method description below. In the SDS gels shown in figure, disappearance of arrestin bands from the supernatant (S) fractions and simultaneous appearance in the membrane fractions (M) indicates positive binding. The binding reactions were carried out in the dark as indicated by ʻ-ʼ (under dim red light conditions), or in light indicated by ʻ+ʼ in the figure.

*Direct binding assay (Method)*. Isolation of rod outer segment (ROS) as well as the subsequent phosphorylation and regeneration of rhodopsin with 11-*cis* retinal was performed as described previously^1^. The concentration of rhodopsin and phosphorylated rhodopsin was determined spectrophotometrically at 498 nm^2^. The centrifugal pull-down assay was used to check the binding of different arrestins to ROS and phosphorylated rhodopsin^3^. Typically, we mixed either phosphorylated or control disk membranes (dark-adapted, containing 100 μM rhodopsin) to one of the purified arrestins (500 nM) in a final volume of 75 μL in binding buffer (10 mM HEPES, 100 mM KCl, pH 7.5) in a transparent centrifuge tube. Samples were incubated either in dark or in ‘light’ (illuminated under a yellow lamp λ ≥ 530 nm) for 10 min. All samples were immediately cooled on ice and centrifuged for 15 min at 50 000 g at 4°C. The clear supernatant and pellet fractions were separated and analysed by SDS-PAGE.

**
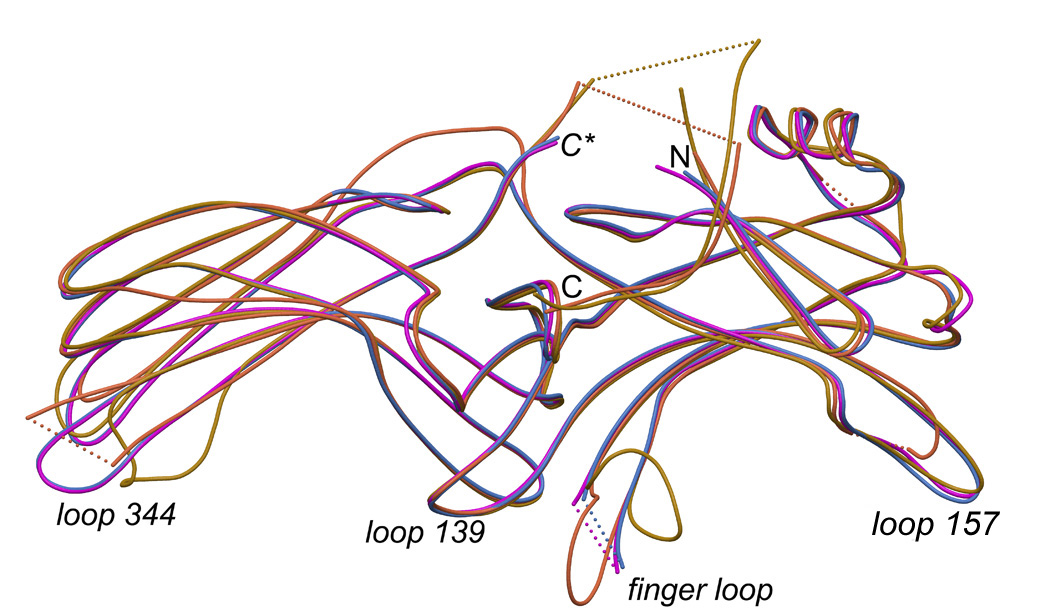
**

**Figure S3.** Superposition of arr-1 structures showing plasticity in the loops (Color code: R175E blue, *PDB ID’s* 3UGX molecule A gold, molecule B coral, 3UGU magenta).


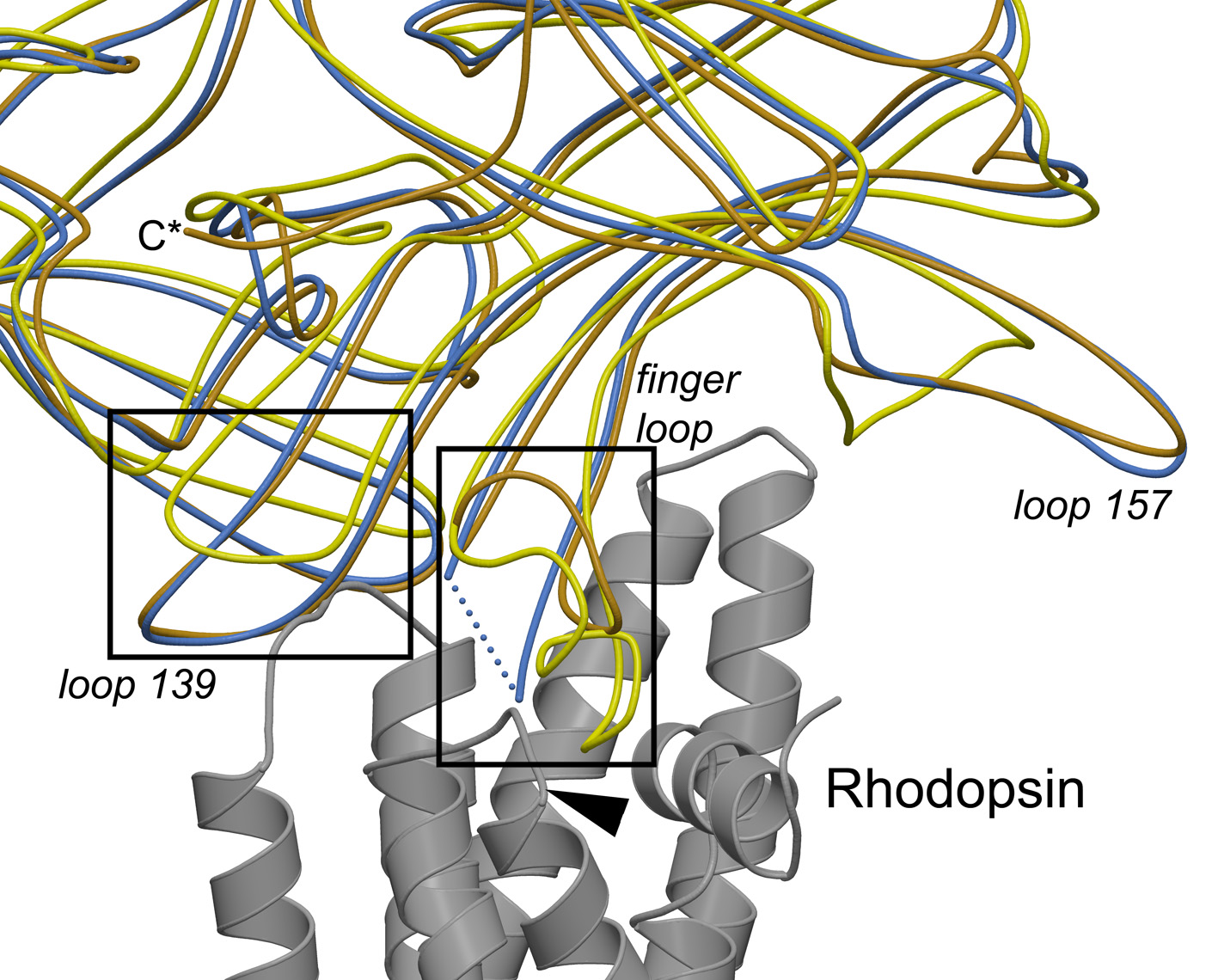


**Figure S4.** Superposition of crystal structures of arr-1, R175E and the rhodopsin-arrestin complex. Highlighted in the boxes are the finger loop and loop 139 (middle loop) (Color code: R175E blue, *PDB ID’s* 3UGX molecule A gold, 4ZWJ with arrestin in yellow and rhodopsin in dark grey). The arrowhead indicates the second intracellular loop (ICL2) of rhodopsin at the interface; C* shows the C-tail terminus in arr-1.

**
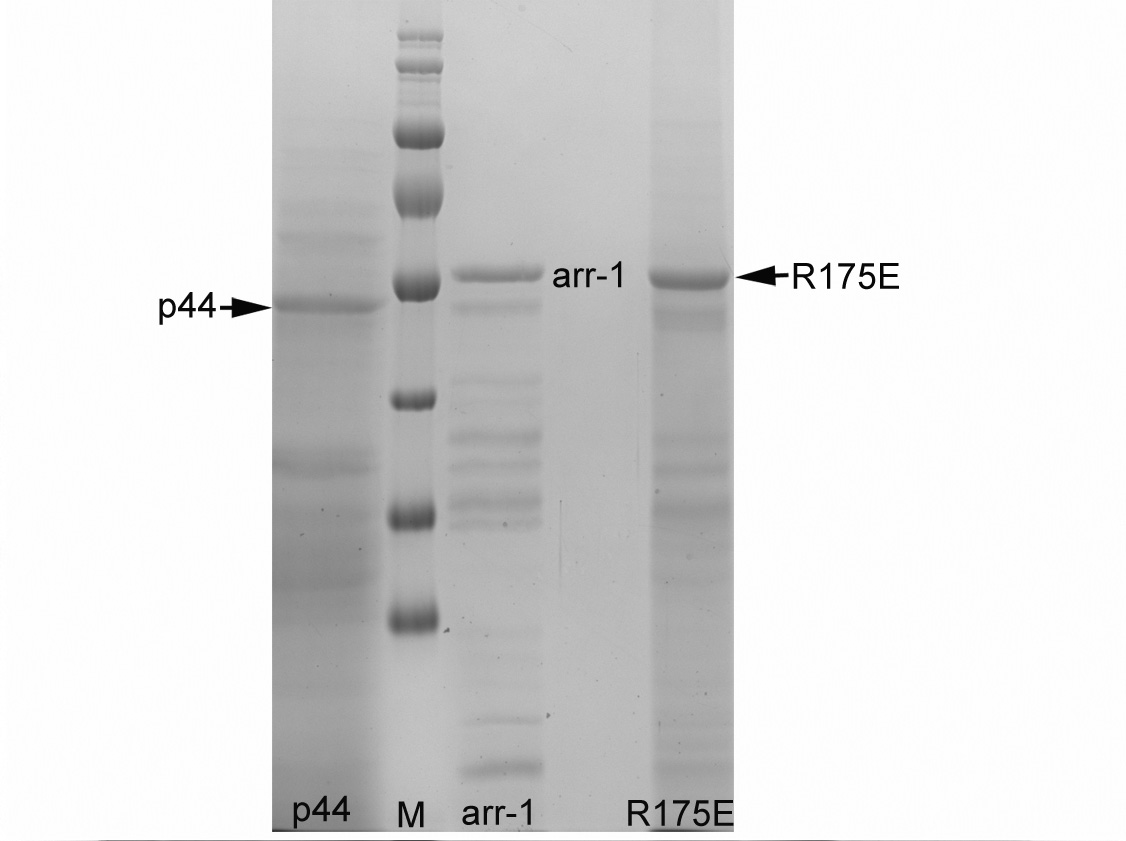
**

**Figure S5.** SDS-gel analysis for the presence of C-terminal residues in R175E crystals. Both, wild type arr-1 and the mutant R175E run at the same height (~50 kDa) whereas the truncated splice variant p44 lacking the C-terminal residues runs at ~44 kDa.

**
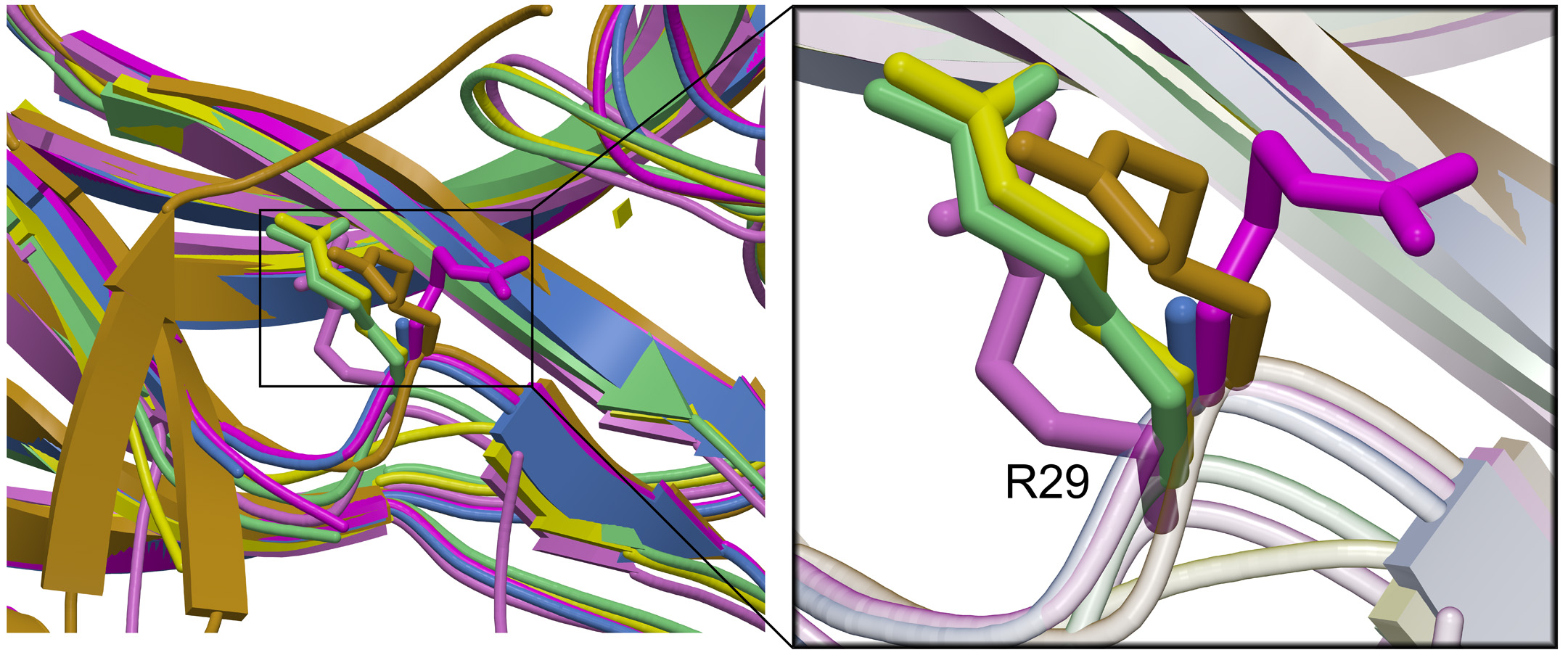
**

**Figure S6.** Superposition of different arrestin structures in basal and active states showing side chain flexibility of residue R29 (Color code: R175E blue, *PDB ID’s* 3UGX gold, 3UGU magenta, 4J2Q green, 4JQI violet, 4ZWJ* yellow). Side chain of R29 in R175E crystal structure is truncated beyond Cβ position due to missing electron density.

*In the crystal structure of rhodopsin-arrestin complex (PDB ID 4ZWJ), residue corresponding to R29 (R2030, numbering in complex) shows no side chain electron density in molecule A and poor density (up to Nε) in molecule B. In contrast, the corresponding residues in molecules C and D show good densities.

**
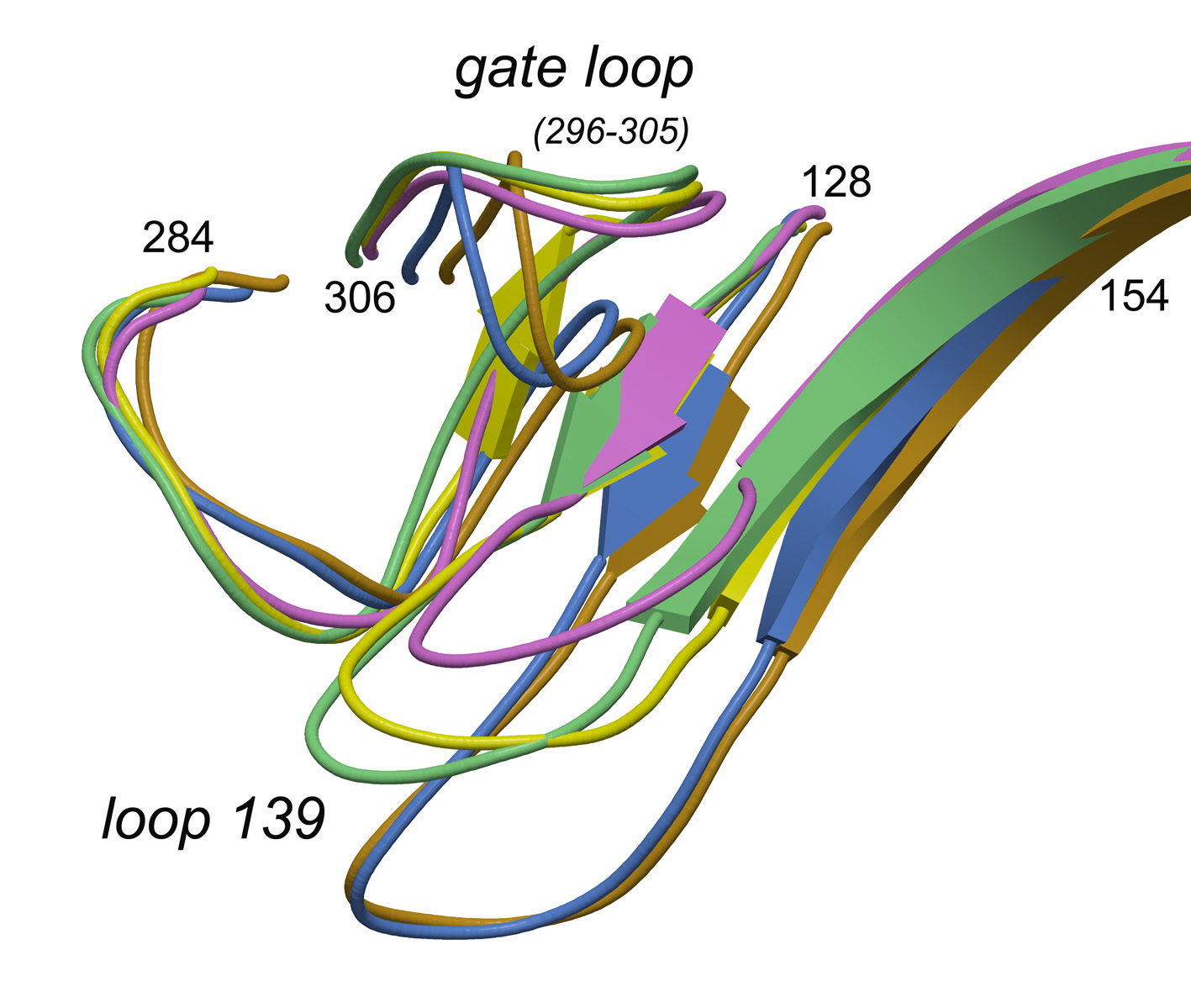
**

**Figure S7.** Superposition of basal and active states of arrestin to show differences in gate loop conformations (Color code: R175E blue, current work; *PDB ID’s* 3UGX gold, basal state; 4J2Q green, p44 with 21° rotation; 4JQI purple, β-arrestin-phosphopeptide complex with 21° rotation; 4ZWJ yellow, rhodopsin-arrestin complex with 20° domain orientation). Additionally, the interaction and thus ‘influence’ between the lariat loop (residues 283-305) and loop 139 is demonstrated.

**
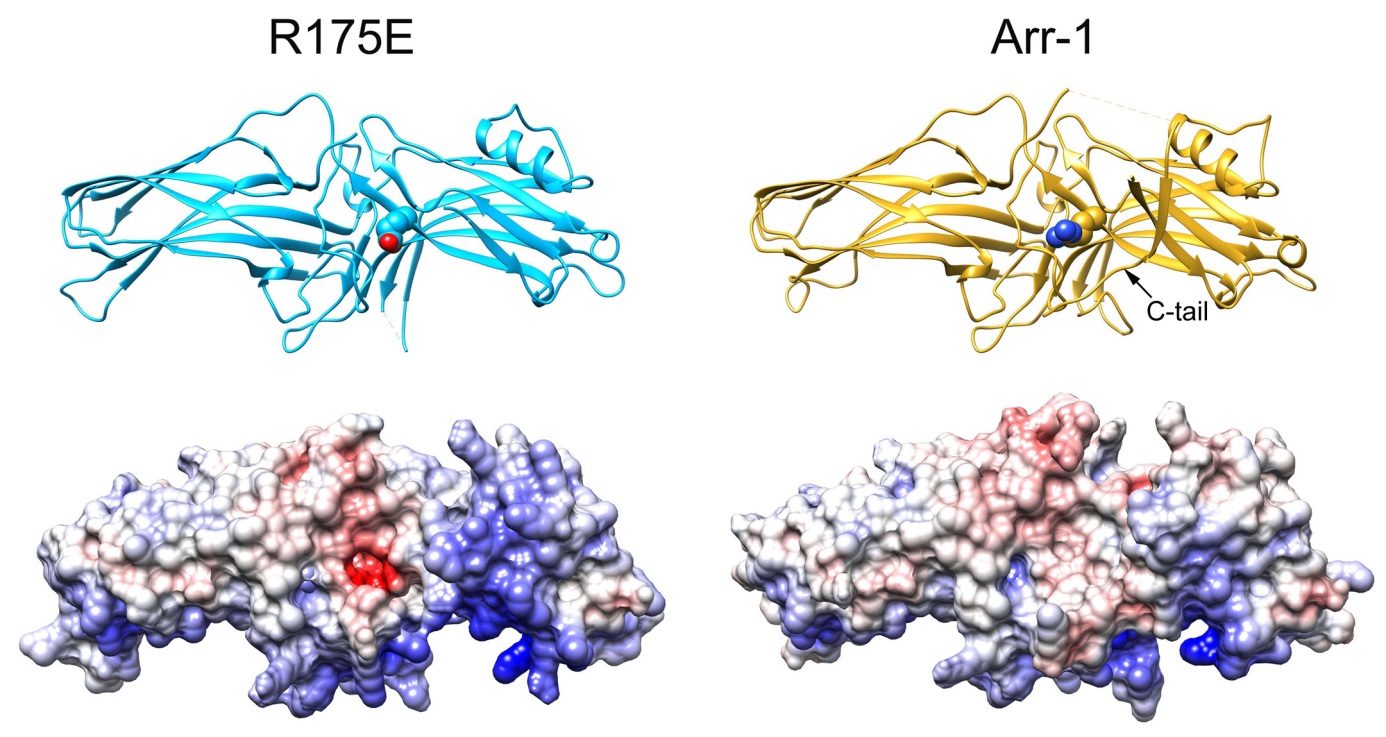
**

**Figure S8.** Electrostatic surfaces of crystal structures of arr-1 and R175E. Comparison of the lower panels shows an increase in overall positive potential in R175E due to exposure of the key phosphor-sensor residues. Electrostatic surface potentials were calculated using APBS^4^ with the nonlinear Poisson-Boltzmann equation and contoured at ± 10 kT/e, negatively and positively charged surface are colored red and blue, respectively. The upper panels are ribbon representations to show the molecule orientation with the residue 175 in the center (Compared to Figure 1, the view is rotated by approximately 45° along the horizontal axis towards the reader).

**Supplementary Tables**

**Table S1.** List of hydrogen bonds with residue 175 (arr-1 numbering) in the polar core region in different arrestin structures deposited in the PDB database (http://www.rcsb.org). The cut-off value for h-bond selection used is ≤3.5 Å.

4ZRG (R175E structure, current work)

Residue No./Chain/Residue name/Atom Bond distance (Å)

175 A GLU N .... 174 A ILE O 2.23

175 A GLU N .... 29 A ARG O 2.78

175 A GLU O .... 176 A LYS N 2.18

175 A GLU O .... 31 A TYR N 3.08

-------------------------------------------------------------

3UGX (basal arr-1)^5^

175 A ARG N .... 29 A ARG O 3.09

175 A ARG N .... 174 A ILE O 2.26

175 A ARG O .... 31 A TYR N 2.80

175 A ARG O .... 176 A LYS N 2.26

175 A ARG NE .... 30 A ASP OD1 2.96

175 A ARG NH1 .... 296 A ASP OD1 2.69

175 A ARG NH1 .... 296 A ASP OD2 3.17

175 A ARG NH1 .... 303 A ASP O 3.10

175 A ARG NH1 .... 305 A ASN ND2 3.35

175 A ARG NH2 .... 303 A ASP O 3.48

175 A ARG NH2 .... 30 A ASP OD2 2.83

175 B ARG N .... 29 B ARG O 3.10

175 B ARG N .... 174 B ILE O 2.26

175 B ARG O .... 31 B TYR N 2.91

175 B ARG O .... 176 B LYS N 2.26

175 B ARG NE .... 30 B ASP OD1 2.72

175 B ARG NH1 .... 303 B ASP O 3.01

175 B ARG NH1 .... 296 B ASP OD1 3.22

175 B ARG NH1 .... 424 B HOH O 2.73

175 B ARG NH2 .... 30 B ASP OD2 3.23

175 B ARG NH2 .... 303 B ASP O 3.32

175 B ARG NH2 .... 420 B HOH O 2.87

175 B ARG NH2 .... 30 B ASP OD1 3.41

175 C ARG N .... 29 C ARG O 3.29

175 C ARG N .... 174 C ILE O 2.25

175 C ARG N .... 176 C LYS N 3.48

175 C ARG O .... 176 C LYS N 2.26

175 C ARG O .... 31 C TYR N 2.84

175 C ARG NE .... 30 C ASP OD1 2.81

175 C ARG NH1 .... 296 C ASP OD1 2.96

175 C ARG NH1 .... 303 C ASP O 2.99

175 C ARG NH2 .... 30 C ASP OD2 2.84

175 D ARG N .... 174 D ILE O 2.27

175 D ARG N .... 29 D ARG O 3.18

175 D ARG O .... 176 D LYS N 2.25

175 D ARG O .... 31 D TYR N 2.89

175 D ARG NE .... 30 D ASP OD1 2.99

175 D ARG NH1 .... 296 D ASP OD1 2.75

175 D ARG NH1 .... 296 D ASP OD2 3.12

175 D ARG NH1 .... 303 D ASP O 3.17

175 D ARG NH2 .... 30 D ASP OD2 2.99

175 D ARG NH2 .... 303 D ASP O 3.27

-------------------------------------------------------------

1CF1 (basal arr-1)^6^

175 A ARG N .... 29 A ARG O 3.19

175 A ARG N .... 174 A ILE O 2.25

175 A ARG O .... 31 A TYR N 2.77

175 A ARG O .... 176 A LYS N 2.24

175 A ARG NE .... 30 A ASP OD1 3.01

175 A ARG NH1 .... 303 A ASP O 2.92

175 A ARG NH1 .... 296 A ASP OD1 2.71

175 A ARG NH1 .... 305 A ASN ND2 3.37

175 A ARG NH2 .... 303 A ASP OD2 3.36

175 A ARG NH2 .... 303 A ASP O 3.49

175 A ARG NH2 .... 30 A ASP OD2 2.90

175 B ARG N .... 29 B ARG O 3.01

175 B ARG N .... 174 B ILE O 2.25

175 B ARG O .... 31 B TYR N 2.77

175 B ARG O .... 176 B LYS N 2.23

175 B ARG NE .... 30 B ASP OD1 2.75

175 B ARG NH1 .... 303 B ASP O 2.96

175 B ARG NH1 .... 296 B ASP OD1 3.01

175 B ARG NH2 .... 30 B ASP OD2 3.08

175 B ARG NH2 .... 30 B ASP OD1 3.38

175 B ARG NH2 .... 303 B ASP OD2 3.29

175 C ARG N .... 174 C ILE O 2.24

175 C ARG N .... 29 C ARG O 3.19

175 C ARG O .... 176 C LYS N 2.25

175 C ARG O .... 31 C TYR N 2.76

175 C ARG NE .... 30 C ASP OD1 3.01

175 C ARG NH1 .... 296 C ASP OD1 2.66

175 C ARG NH1 .... 303 C ASP O 2.89

175 C ARG NH1 .... 305 C ASN ND2 3.38

175 C ARG NH2 .... 303 C ASP O 3.41

175 C ARG NH2 .... 30 C ASP OD2 2.92

175 C ARG NH2 .... 303 C ASP OD2 3.28

175 D ARG N .... 174 D ILE O 2.25

175 D ARG N .... 29 D ARG O 2.98

175 D ARG O .... 176 D LYS N 2.23

175 D ARG O .... 31 D TYR N 2.77

175 D ARG NE .... 30 D ASP OD1 2.74

175 D ARG NH1 .... 303 D ASP O 3.02

175 D ARG NH1 .... 296 D ASP OD1 3.02

175 D ARG NH2 .... 30 D ASP OD1 3.38

175 D ARG NH2 .... 30 D ASP OD2 3.06

175 D ARG NH2 .... 303 D ASP OD2 3.27

-------------------------------------------------------------

3UGU (p44)^5^

175 A ARG N .... 174 A ILE O 2.24

175 A ARG N .... 29 A ARG O 2.84

175 A ARG O .... 176 A LYS N 2.25

175 A ARG O .... 31 A TYR N 2.77

175 A ARG NE .... 29 A ARG NH1 2.74

175 A ARG NE .... 30 A ASP OD1 2.94

175 A ARG NH1 .... 296 A ASP OD1 3.00

175 A ARG NH1 .... 296 A ASP OD2 3.49

175 A ARG NH1 .... 492 A HOH O 3.14

175 A ARG NH1 .... 296 A ASP O 3.05

175 A ARG NH2 .... 492 A HOH O 3.07

175 A ARG NH2 .... 30 A ASP OD2 2.93

-------------------------------------------------------------

4J2Q (p44 with 21 ° domain orientation)^7^

175 A ARG N .... 29 A ARG O 2.90

175 A ARG N .... 174 A ILE O 2.26

175 A ARG O .... 31 A TYR N 2.78

175 A ARG O .... 176 A LYS N 2.27

175 A ARG NE .... 30 A ASP OD1 2.77

175 A ARG NH1 .... 297 A GLY O 2.75

175 A ARG NH2 .... 30 A ASP OD2 3.29

175 A ARG NH2 .... 30 A ASP OD1 3.14

175 B ARG N .... 29 B ARG O 3.11

175 B ARG N .... 174 B ILE O 2.27

175 B ARG O .... 176 B LYS N 2.27

175 B ARG O .... 31 B TYR N 2.78

175 B ARG NE .... 30 B ASP OD1 3.42

175 B ARG NE .... 297 B GLY O 2.79

175 B ARG NH1 .... 30 B ASP OD1 2.97

175 B ARG NH2 .... 297 B GLY O 3.42

-------------------------------------------------------------

4JQI (β-arrestin-peptide complex with 21 ° domain orientation)^8^

169 A ARG N .... 25 A ARG O 3.17

169 A ARG N .... 168 A ILE O 2.28

169 A ARG O .... 27 A PHE N 2.90

169 A ARG O .... 170 A LYS N 2.25

169 A ARG NE .... 26 A ASP OD1 2.89

169 A ARG NH1 .... 291 A GLY O 3.25

169 A ARG NH2 .... 26 A ASP OD2 2.83

169 A ARG NH2 .... 26 A ASP OD1 3.08

------------------------------------------------------------

4ZWJ (rhodopsin-arrestin complex with with 20 ° domain orientation)^9^

2176 A ARG N .... 2175 A ILE O 2.36

2176 A ARG N .... 2030 A ARG O 3.19

2176 A ARG O .... 2032 A TYR O 2.50

2176 A ARG O .... 2177 A LYS N 2.26

2176 A ARG O .... 2032 A TYR N 3.04

2176 A ARG NE .... 2031 A ASP OD1 3.47

2176 A ARG NH1 .... 2298 A GLY O 2.67

2176 A ARG NH2 .... 2031 A ASP OD2 2.88

2176 B ARG N .... 2030 B ARG O 3.22

2176 B ARG N .... 2175 B ILE O 2.36

2176 B ARG O .... 2032 B TYR O 2.48

2176 B ARG O .... 2177 B LYS N 2.26

2176 B ARG O .... 2032 B TYR N 3.03

2176 B ARG NE .... 2031 B ASP OD1 3.44

2176 B ARG NH1 .... 2298 B GLY O 2.68

2176 B ARG NH2 .... 2031 B ASP OD2 2.83

2176 C ARG N .... 2030 C ARG O 3.19

2176 C ARG N .... 2175 C ILE O 2.36

2176 C ARG O .... 2032 C TYR N 3.04

2176 C ARG O .... 2032 C TYR O 2.48

2176 C ARG O .... 2177 C LYS N 2.26

2176 C ARG NE .... 2031 C ASP OD1 3.47

2176 C ARG NH1 .... 2298 C GLY O 2.69

2176 C ARG NH2 .... 2031 C ASP OD2 2.88

2176 D ARG N .... 2030 D ARG O 3.13

2176 D ARG N .... 2175 D ILE O 2.36

2176 D ARG O .... 2032 D TYR O 2.49

2176 D ARG O .... 2177 D LYS N 2.26

2176 D ARG O .... 2032 D TYR N 3.01

2176 D ARG NE .... 2031 D ASP OD1 3.42

2176 D ARG NH1 .... 2298 D GLY O 2.66

2176 D ARG NH2 .... 2031 D ASP OD2 2.83

------------------------------------------------------------

3P2D (arrestin 3)^10^

170 A ARG N .... 169 A ILE O 2.26

170 A ARG N .... 26 A ARG O 3.37

170 A ARG O .... 171 A LYS N 2.25

170 A ARG O .... 28 A PHE N 2.80

170 A ARG NE .... 27 A ASP OD1 3.08

170 A ARG NH1 .... 291 A ASP OD1 3.16

170 A ARG NH1 .... 291 A ASP OD2 3.35

170 A ARG NH1 .... 298 A ASP O 2.98

170 A ARG NH2 .... 298 A ASP O 3.38

170 A ARG NH2 .... 27 A ASP OD2 2.91

170 B ARG N .... 169 B ILE O 2.25

170 B ARG O .... 171 B LYS N 2.28

170 B ARG O .... 28 B PHE N 2.72

170 B ARG NE .... 291 B ASP O 3.37

170 B ARG NE .... 27 B ASP OD2 3.46

170 B ARG NH1 .... 291 B ASP O 3.03

170 B ARG NH1 .... 291 B ASP OD1 3.40

170 B ARG NH1 .... 298 B ASP O 3.14

170 B ARG NH2 .... 298 B ASP O 2.86

170 B ARG NH2 .... 27 B ASP OD2 2.80

-------------------------------------------------------------

1SUJ (cone arrestin)^11^

166 A ARG N .... 24 A ARG O 3.17

166 A ARG N .... 165 A ILE O 2.23

166 A ARG O .... 26 A PHE N 2.80

166 A ARG O .... 26 A PHE O 3.48

166 A ARG O .... 167 A LYS N 2.25

166 A ARG NE .... 25 A ASP OD1 2.66

166 A ARG NH1 .... 287 A ASP OD2 3.08

166 A ARG NH1 .... 287 A ASP OD1 3.10

166 A ARG NH1 .... 294 A ASP O 2.69

166 A ARG NH2 .... 25 A ASP OD1 3.01

166 A ARG NH2 .... 294 A ASP OD2 3.44

166 A ARG NH2 .... 427 A HOH O 3.24

166 A ARG NH2 .... 25 A ASP OD2 2.59

-------------------------------------------------------------

1G4M (bovine β-arrestin 1)^12^

169 A ARG N .... 168 A ILE O 2.25

169 A ARG N .... 25 A ARG O 2.96

169 A ARG O .... 27 A PHE N 2.68

169 A ARG O .... 170 A LYS N 2.29

169 A ARG NE .... 26 A ASP OD1 2.89

169 A ARG NH1 .... 290 A ASP O 2.97

169 A ARG NH1 .... 490 A HOH O 3.13

169 A ARG NH1 .... 290 A ASP OD1 2.85

169 A ARG NH2 .... 26 A ASP OD1 3.42

169 A ARG NH2 .... 297 A ASP OD2 3.44

169 A ARG NH2 .... 297 A ASP O 3.44

169 A ARG NH2 .... 26 A ASP OD2 2.91

169 B ARG N .... 25 B ARG O 2.94

169 B ARG N .... 168 B ILE O 2.25

169 B ARG O .... 170 B LYS N 2.25

169 B ARG O .... 27 B PHE N 2.85

169 B ARG NE .... 26 B ASP OD1 2.85

169 B ARG NH1 .... 290 B ASP OD1 2.82

169 B ARG NH1 .... 290 B ASP OD2 3.45

169 B ARG NH1 .... 290 B ASP O 3.07

169 B ARG NH2 .... 297 B ASP O 3.46

169 B ARG NH2 .... 297 B ASP OD2 3.17

169 B ARG NH2 .... 26 B ASP OD2 2.82

169 B ARG NH2 .... 26 B ASP OD1 3.49

-------------------------------------------------------------

1G4R (bovine β-arrestin 1)^12^

169 A ARG N .... 168 A ILE O 2.25

169 A ARG N .... 25 A ARG O 3.01

169 A ARG O .... 27 A PHE O 3.42

169 A ARG O .... 170 A LYS N 2.24

169 A ARG O .... 27 A PHE N 2.83

169 A ARG NE .... 26 A ASP OD1 2.88

169 A ARG NH1 .... 290 A ASP OD1 2.73

169 A ARG NH1 .... 290 A ASP OD2 3.24

169 A ARG NH1 .... 297 A ASP O 3.23

169 A ARG NH2 .... 26 A ASP OD2 2.83

169 A ARG NH2 .... 26 A ASP OD1 3.45

-------------------------------------------------------------

1JSY (arrestin 2)^13^

169 A ARG N .... 168 A ILE O 2.24

169 A ARG N .... 25 A ARG O 3.09

169 A ARG O .... 27 A PHE N 2.68

169 A ARG O .... 27 A PHE O 3.33

169 A ARG O .... 170 A LYS N 2.24

169 A ARG NE .... 26 A ASP OD1 3.35

169 A ARG NE .... 26 A ASP OD2 3.41

169 A ARG NH1 .... 297 A ASP O 3.15

169 A ARG NH1 .... 290 A ASP O 3.32

169 A ARG NH2 .... 297 A ASP O 3.07

169 A ARG NH2 .... 26 A ASP OD1 2.47

-------------------------------------------------------------

3GC3 (arrestin 2–clathrin complex)^14^

169 A ARG N .... 168 A ILE O 2.24

169 A ARG N .... 25 A ARG O 2.78

169 A ARG O .... 170 A LYS N 2.28

169 A ARG O .... 27 A PHE N 2.60

169 A ARG NE .... 26 A ASP OD1 2.93

169 A ARG NH1 .... 290 A ASP O 2.92

169 A ARG NH1 .... 290 A ASP OD1 2.91

169 A ARG NH1 .... 493 A HOH O 3.15

169 A ARG NH2 .... 26 A ASP OD2 2.68

169 A ARG NH2 .... 26 A ASP OD1 3.30

169 A ARG NH2 .... 297 A ASP O 3.44

-------------------------------------------------------------

3GD1 (arrestin 2 –clathrin long isoform)^14^

169 C ARG N .... 25 C ARG O 3.03

169 C ARG N .... 168 C ILE O 2.25

169 C ARG O .... 27 C PHE N 2.83

169 C ARG O .... 170 C LYS N 2.25

169 C ARG NE .... 26 C ASP OD1 2.82

169 C ARG NH1 .... 290 C ASP O 3.28

169 C ARG NH1 .... 290 C ASP OD1 2.95

169 C ARG NH1 .... 297 C ASP O 3.19

169 C ARG NH1 .... 297 C ASP OD1 3.17

169 C ARG NH2 .... 26 C ASP OD2 3.40

169 C ARG NH2 .... 297 C ASP O 3.39

169 E ARG N .... 168 E ILE O 2.26

169 E ARG O .... 170 E LYS N 2.24

169 E ARG O .... 27 E PHE N 3.16

169 E ARG NH1 .... 290 E ASP OD1 2.98

169 E ARG NH1 .... 297 E ASP O 3.11

169 E ARG NH2 .... 298 E THR OG1 3.27

169 E ARG NH2 .... 297 E ASP O 3.45

169 E ARG NH2 .... 298 E THR N 2.83

-------------------------------------------------------------

2WTR (β-arrestin 1) (*unpublished*)

169 A ARG N .... 168 A ILE O 2.25

169 A ARG O .... 170 A LYS N 2.25

169 A ARG O .... 27 A PHE N 2.87

169 A ARG NE .... 393 A ARG NH2 2.91

169 A ARG NH1 .... 290 A ASP O 3.26

169 A ARG NH1 .... 290 A ASP OD1 3.27

169 A ARG NH2 .... 26 A ASP OD2 3.33

169 A ARG NH2 .... 297 A ASP O 3.27

169 B ARG N .... 25 B ARG O 3.23

169 B ARG N .... 168 B ILE O 2.25

169 B ARG O .... 27 B PHE N 2.79

169 B ARG O .... 170 B LYS N 2.25

169 B ARG O .... 27 B PHE O 3.36

169 B ARG NE .... 26 B ASP OD1 2.94

169 B ARG NH2 .... 26 B ASP OD2 2.90

-------------------------------------------------------------

**Table S2.** List of hydrogen bonds involving C-tail residues (372-386) in basal state arr-1 showing interaction with residues from the polar core and three-element regions. The cut-off criterion is ≤3.5 Å.

*PDB ID* 3UGX (molecule A)

Residue No./Chain/Residue name/Atom H-bond length in Å

372 A ASP N .... 373 A GLU N 3.25

372 A ASP O .... 373 A GLU N 2.25

372 A ASP OD1 .... 373 A GLU N 3.12

372 A ASP OD2 .... 106 A SER OG 3.27

373 A GLU N .... 372 A ASP O 2.25

373 A GLU N .... 372 A ASP N 3.25

373 A GLU N .... 372 A ASP OD1 3.12

373 A GLU O .... 374 A ASN N 2.25

374 A ASN N .... 373 A GLU O 2.25

374 A ASN O .... **10** A HIS N 2.81

374 A ASN O .... 375 A PHE N 2.25

374 A ASN ND2 .... **9** A ASN OD1 2.45

375 A PHE N .... 374 A ASN O 2.25

375 A PHE O .... 376 A VAL N 2.26

376 A VAL N .... **10** A HIS O 2.90

376 A VAL N .... 375 A PHE O 2.26

376 A VAL N .... 377 A PHE N 3.40

376 A VAL O .... **12** A ILE N 2.87

376 A VAL O .... 377 A PHE N 2.25

377 A PHE N .... 376 A VAL N 3.40

377 A PHE N .... 376 A VAL O 2.25

377 A PHE N .... 378 A GLU N 3.48

377 A PHE O .... 378 A GLU N 2.25

377 A PHE O .... 409 A PTD O1 3.25

378 A GLU N .... 377 A PHE N 3.48

378 A GLU N .... **12** A ILE O 2.88

378 A GLU N .... 377 A PHE O 2.25

378 A GLU O .... 379 A GLU O 3.50

378 A GLU O .... **12** A ILE O 3.34

378 A GLU O .... 379 A GLU N 2.27

378 A GLU O .... **14** A LYS N 2.94

378 A GLU OE2 .... **29** A ARG NH2 3.12

379 A GLU N .... 378 A GLU O 2.27

379 A GLU O .... **29** A ARG NH2 3.38

379 A GLU O .... 381 A ALA N 3.42

379 A GLU O .... 378 A GLU O 3.50

379 A GLU O .... 380 A PHE N 2.25

380 A PHE N .... 379 A GLU O 2.25

380 A PHE N .... 381 A ALA N 2.76

380 A PHE N .... **14** A LYS O 3.12

380 A PHE O .... **29** A ARG NH2 3.48

380 A PHE O .... **29** A ARG NE 2.64

380 A PHE O .... 381 A ALA O 3.20

380 A PHE O .... 381 A ALA N 2.25

380 A PHE O .... 382 A ARG N 3.26

381 A ALA N .... 379 A GLU O 3.42

381 A ALA N .... 380 A PHE O 2.25

381 A ALA N .... 380 A PHE N 2.76

381 A ALA O .... 380 A PHE O 3.20

381 A ALA O .... 382 A ARG N 2.26

382 A ARG N .... 381 A ALA O 2.26

382 A ARG N .... 380 A PHE O 3.26

382 A ARG O .... 384 A ASN N 3.48

382 A ARG O .... 383 A GLN N 2.27

382 A ARG NH1 .... **30** A ASP OD1 3.24

382 A ARG NH1 .... 303 A ASP OD2 2.59

382 A ARG NH2 .... **29** A ARG O 3.27

382 A ARG NH2 .... **173** A LEU O 3.32

383 A GLN N .... 382 A ARG O 2.27

383 A GLN O .... 302 A GLU N 2.97

383 A GLN O .... 384 A ASN N 2.26

383 A GLN O .... 384 A ASN O 3.43

384 A ASN N .... 383 A GLN O 2.26

384 A ASN N .... 382 A ARG O 3.48

384 A ASN O .... 385 A LEU O 3.42

384 A ASN O .... 386 A LYS N 3.39

384 A ASN O .... 383 A GLN O 3.43

384 A ASN O .... 385 A LEU N 2.26

385 A LEU N .... 300 A LYS O 2.53

385 A LEU N .... 384 A ASN O 2.26

385 A LEU O .... 386 A LYS N 2.26

385 A LEU O .... 384 A ASN O 3.42

385 A LEU O .... 386 A LYS O 3.09

386 A LYS N .... 385 A LEU O 2.26

386 A LYS N .... 384 A ASN O 3.39

386 A LYS O .... 385 A LEU O 3.09

-------------------------------------------------------------

**Table S3.** Size exclusion chromatography of arr-1, R175E and p44 arrestins using Superdex 200 10/300 GL column.

| Protein | Elution volume* (Ve) mL | Apparent mass (kDa) | Calculated mass (kDa) |
| --- | --- | --- | --- |
| Arr-1  p44  R175E | 14.6  15.36  14.97 | 62.9  39.9  47.11 | 47.1  42.2  47.1 |

* Average of two runs

**References to Supplementary Information**

1 Pulvermuller, A., Schroder, K., Fischer, T. & Hofmann, K. P. Interactions of metarhodopsin II - Arrestin peptides compete with arrestin and transducin. *J. Biol. Chem.* **275**, 37679-37685 (2000).

2 Wald, G. & Brown, P. K. The Molar Extinction of Rhodopsin. *J. Gen. Physiol.* **37**, 189-200 (1953).

3 Kuhn, H., Hall, S. W. & Wilden, U. Light-Induced Binding of 48-Kda Protein to Photoreceptor-Membranes Is Highly Enhanced by Phosphorylation of Rhodopsin. *Febs Lett.* **176**, 473-478 (1984).

4 Baker, N. A., Sept, D., Joseph, S., Holst, M. J. & McCammon, J. A. Electrostatics of nanosystems: Application to microtubules and the ribosome. *P. Natl. Acad. Sci. USA* **98**, 10037-10041 (2001).

5 Granzin, J. *et al.* Crystal Structure of p44, a Constitutively Active Splice Variant of Visual Arrestin. *J. Mol. Biol.* **416**, 611-618 (2012).

6 Hirsch, J. A., Schubert, C., Gurevich, V. V. & Sigler, P. B. The 2.8 angstrom crystal structure of visual arrestin: A model for arrestin's regulation. *Cell* **97**, 257-269 (1999).

7 Kim, Y. J. *et al.* Crystal structure of pre-activated arrestin p44. *Nature* **497**, 142-146 (2013).

8 Shukla, A. K. *et al.* Structure of active beta-arrestin-1 bound to a G-protein-coupled receptor phosphopeptide. *Nature* **497**, 137-141 (2013).

9 Kang, Y. *et al*. Crystal structure of rhodopsin bound to arrestin by femtosecond X-ray laser. *Nature* **523**, 561-567 (2015).

10 Zhan, X. Z., Gimenez, L. E., Gurevich, V. V. & Spiller, B. W. Crystal Structure of Arrestin-3 Reveals the Basis of the Difference in Receptor Binding Between Two Non-visual Subtypes. *J. Mol. Biol.* **406**, 467-478 (2011).

11 Sutton, R. B. *et al.* Crystal structure of cone arrestin at 2.3A: evolution of receptor specificity. *J. Mol. Biol.* **354**, 1069-1080 (2005).

12 Han, M., Gurevich, V. V., Vishnivetskiy, S. A., Sigler, P. B. & Schubert, C. Crystal structure of beta-arrestin at 1.9 angstrom: Possible mechanism of receptor binding and membrane translocation. *Structure* **9**, 869-880 (2001).

13 Milano, S. K., Pace, H. C., Kim, Y. M., Brenner, C. & Benovic, J. L. Scaffolding functions of arrestin-2 revealed by crystal structure and mutagenesis. *Biochemistry-Us* **41**, 3321-3328 (2002).

14 Kang, D. S. *et al.* Structure of an Arrestin2-Clathrin Complex Reveals a Novel Clathrin Binding Domain That Modulates Receptor Trafficking. *J. Biol. Chem.* **284**, 29860-29872 (2009).
